# Modeling the Viscoelastic Hysteresis of Dielectric Elastomer Actuators with a Modified Rate-Dependent Prandtl–Ishlinskii Model

**DOI:** 10.3390/polym10050525

**Published:** 2018-05-14

**Authors:** Jiang Zou, Guoying Gu

**Affiliations:** 1 State Key Laboratory of Mechanical System and Vibration, School of Mechanical Engineering, Shanghai Jiao Tong University, Shanghai 200240, China; sj_ustb@sjtu.edu.cn; 2 Robotics Institute, School of Mechanical Engineering, Shanghai Jiao Tong University, Shanghai 200240, China

**Keywords:** dielectric elastomer actuators, viscoelastic hysteresis nonlinearity, phenomenal modeling approach, modified Prandtl–Ishlinskii model

## Abstract

Dielectric elastomer actuators (DEAs) are known as a type of electric-driven artificial muscle that have shown promising potential in the field of soft robotics. However, the inherent viscoelastic nonlinearity makes the modeling and control of DEAs challenging. In this paper, we propose a new phenomenological modeling approach with the Prandtl–Ishlinskii (P–I) model to characterize the viscoelastic hysteresis nonlinearity of DEAs. Differently from the commonly used physics-based models, the developed phenomenological model, called the modified rate-dependent P–I model (MRPIM), produces behavior similar to that of physics-based models but without necessarily considering physical insight into the modeling problem. In this way, the developed MRPIM can characterize the asymmetric and rate-dependent viscoelastic hysteresis with a relative simple mathematical format using only the experimental data. To validate the development, experimental tests were conducted with seven different frequencies; four were selected to identify the model parameters and the rest of the data were used to further verify the model. Comparisons between the model prediction and experimental data demonstrate that the MRPIM can precisely describe the viscoelastic hysteresis effect of DEAs with a maximum prediction error of 9.03% and root-mean-square prediction error of 4.50%.

## 1. Introduction

Dielectric elastomer actuators (DEAs) are a promising actuation technology for soft robotics, owing to the advantages of large strain (>380%), high energy density (>3.4 MJ/m^3^), and fast response (in the order of milliseconds) [1,2]. The working principle of DEAs is simple: under an applied electric field, the Maxwell stress squeezes the elastomer membrane in thickness and leads to expansion in area while keeping a constant volume [3]. On the basis of this principle, many DEA configurations have been invented to generate different degree-of-freedom (DOF) motions, such as rectilinear motion [4], rotational motion [5], and bending motion [6]. It is also interesting to find that these motions can mimic natural muscle, which makes them promising as artificial muscles for bioinspired soft robots, for instance, an arm wrestling robot [7], two locomotive robots [8,9], and a swimming fish [10].

**Figure 1:**
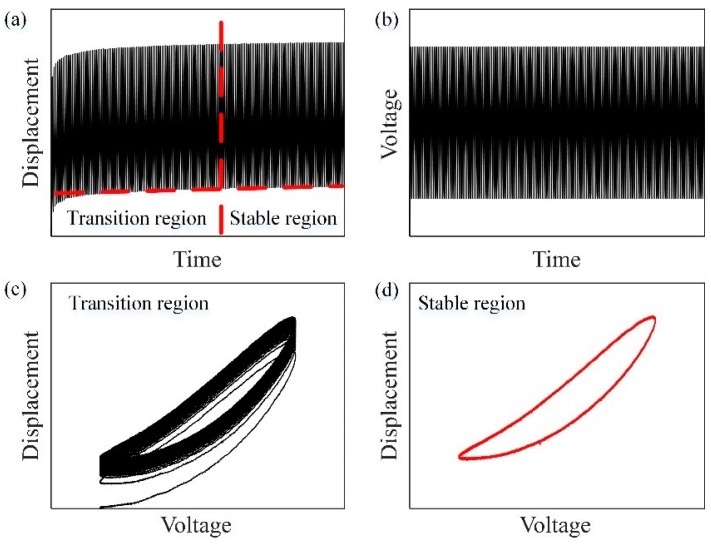
Experimental response of a dielectric elastomer actuator (DEA) under a sinusoidal exciting voltage: (**a**) the output displacement; (**b**) the sinusoidal exciting voltage; (**c**) the viscoelastic creep effect during transition region; (**d**) the viscoelastic hysteresis effect in stable region.

Despite the diverse achievements in mechanism design, there still remains a challenge to accurately characterize the response of DEAs because of their inherent viscoelasticity. Figure 1a illustrates an experimental response of a DEA under a sinusoidal exciting input (Figure 1b). From the results, we can observe that the response can be divided into two regions: a transition region and a stable region. In the transition region, the output displacement creeps over extended periods of time, as shown in Figure 1c, where the displacement decreases over time and becomes less dominant after a few cycles [11]. Many well-defined models [12,13,14] have been developed to describe this viscoelastic creep effect. It should be noted that, even without a creep model [15], a conventional feedback controller can easily mitigate such creep effect. On the other hand, viscoelastic hysteresis effect dominates the response of DEAs during the stable region (Figure 1d). Hysteresis is a non-smooth nonlinearity with multivalued and nonlocal memoryless behavior [16,17]. It can significantly degrade the performance of actuators, which leads, in the best case, to a reduction in the motion accuracy and, in the worst case, to destabilization of the control system [18,19,20,21,22]. By measuring the relationship between displacement, force, and input voltage, York et al. [23] recently pointed out that the hysteresis mainly causes positioning inaccuracy of DEAs. Therefore, it is important and necessary to understand and characterize the hysteretic nonlinearity of DEAs.

To address this challenge, many efforts have been made in the literature. In general, the viscoelastic hysteresis can be considered as one of several dissipative phenomena, and some physics-based models [11,24,25,26] have been established to explain it. A main advantage of such physics-based models depends on the fact that they are built on the basis of understanding the physical behaviors of DEAs. However, they are generally very complicated in order to describe the experimental phenomena. For example, the creep effect was explained by a Gent model with 8 material parameters [27], but 20 parameters should be used to describe the creep, hysteresis, and the shift in the displacement’s peak when the DEA is subjected to a cycle loading with a constant slope [11]. In addition, when the slope is variable, the model will be more complicated.

Rather than using physics-based models, some phenomenological models have been reported to describe the viscoelastic hysteresis effect of DEAs by using only experimental data without considering their physical behaviors. For example, by linearizing the input voltage, the hysteresis loops of a tubular DEA became symmetric and ellipse-like, and an elliptical modeling method was used to describe the hysteresis effect [28]. In addition, a polynomial-based approach was developed by using two polynomials to model the increasing and decreasing hysteresis loops [29].

However, the drawbacks of these existing phenomenological models are clear: they are either rate-independent or it is necessary to change the model parameters for a rate-dependent hysteresis description. It is known that the P–I model is a popular phenomenological model to describe the complex hysteresis nonlinearity of smart material actuators [16,17,18,19,22], but less attention has been paid to employ the P–I model for the viscoelastic hysteresis description of DEAs.

As a first attempt, this paper presents a modified rate-dependent P–I model (MRPIM) to describe the asymmetric and rate-dependent viscoelastic hysteresis effect of a DEA. The developed MRPIM consists of three parts: (i) a fourth-order polynomial is introduced to describe the asymmetric behavior; (ii) fixed weights and thresholds of the play operators with dynamical envelope functions are used to characterize the rate-dependent behavior; (iii) the second-order derivative of the input voltage is introduced to the fourth-order polynomial for describing the peak-to-peak displacements of the hysteresis loops that highly depend on the frequency of the input voltage. The advantages of our model lie in the facts that (1) it can precisely describe the viscoelastic hysteresis of DEAs with only experimental data; (2) compared with physics-based models, there are relatively fewer parameters to describe both asymmetric and rate-dependent hysteresis behavior of DEAs; and (3) differently from existing phenomenological models [28,29], our model is rate-dependent and only needs one step to identify all the parameters without data pre-processing. In order to verify the effectiveness of the MRPIM, a planar DEA was fabricated, and experiments were conducted. Experimental results demonstrate that the MRPIM can accurately predict the hysteresis effect of the DEA with different frequencies (in the range of 0.02 to 1 Hz). The maximum prediction error and root-mean-square prediction error were under 9.03% and 4.50%, respectively. In addition, it should be noted that our MRPIM can be used not only to describe the hysteresis effect of the planer DEA, but also can be applied for different DEAs (e.g., [28,29]) that have asymmetric and rate-dependent viscoelastic hysteresis behavior. The only difference depends on the model parameters, which need to be identified on the basis of experimental data of different DEAs.

The remainder of this paper is arranged as follows. Section 2 describes our fabricated planar DEA and the experimental setup; the viscoelastic hysteresis effect of DEAs is also analyzed in this section. Section 3 introduces the development, identification, and verification of the MRPIM. Finally, Section 4 concludes this paper.

## 2. System Description

### 2.1. Fabrication of the Planar DEA

**Figure 2:**
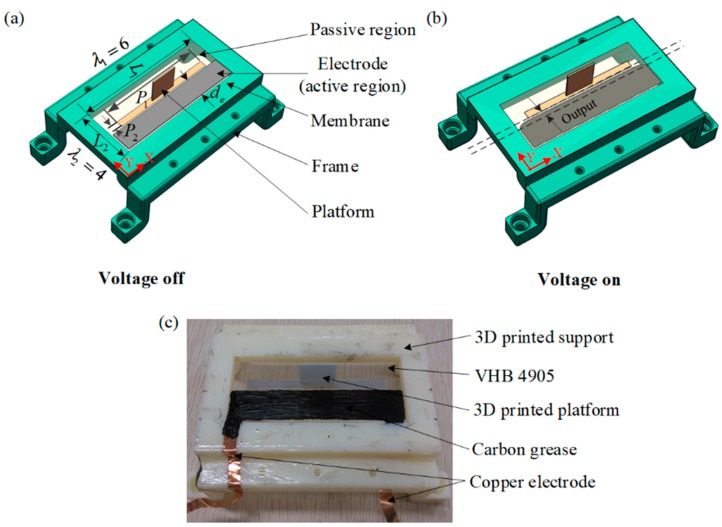
Description of the designed planar dielectric elastomer actuator (DEA): (**a**) the mechanism structure; (**b**) working principle; (**c**) a picture of one fabricated DEA.

In this work, a planar DEA as shown in Figure 2a was fabricated for proof-of-concept examining. The fabrication processes can be summarized as follows: (i) A prestrained DE membrane (3M VHB 4905, $L_{1}\times L_{2}=100\;\mathrm{mm}\times 40\;\mathrm{mm}$, $\lambda_{1}\times \lambda_{2}\;=\;6\times 4$) is supported by a 3D printed frame. (ii) A platform ($P_{1}\times P_{2}=80\;\mathrm{mm}\times 5\;\mathrm{mm}$) is fixed on the middle of the DE membrane to separate the DE membrane into passive and active regions. (iii) Carbon grease is used as electrodes covering both sides of the active region (the right part of the DE membrane). It should be noted that in order to avoid electric break down, there is an interval of 1 mm between the electrodes and the edges of other parts, such that each electrode is a rectangular area with a length of 98 mm and a width of 15.5 mm. (iv) The electrodes are connected to a high-voltage source.

The planar DEA can provide a linear actuation with one DOF. As shown in Figure 2b, its working principle can be described as follows: when a voltage is applied to the electrodes, the active region will expand along the Y-direction and the passive region works as a nonlinear spring [30,31], such that the platform will generate an output displacement. Figure 2c shows a photo of the fabricated planar DEA.

### 2.2. Experimental Setup

**Figure 3:**
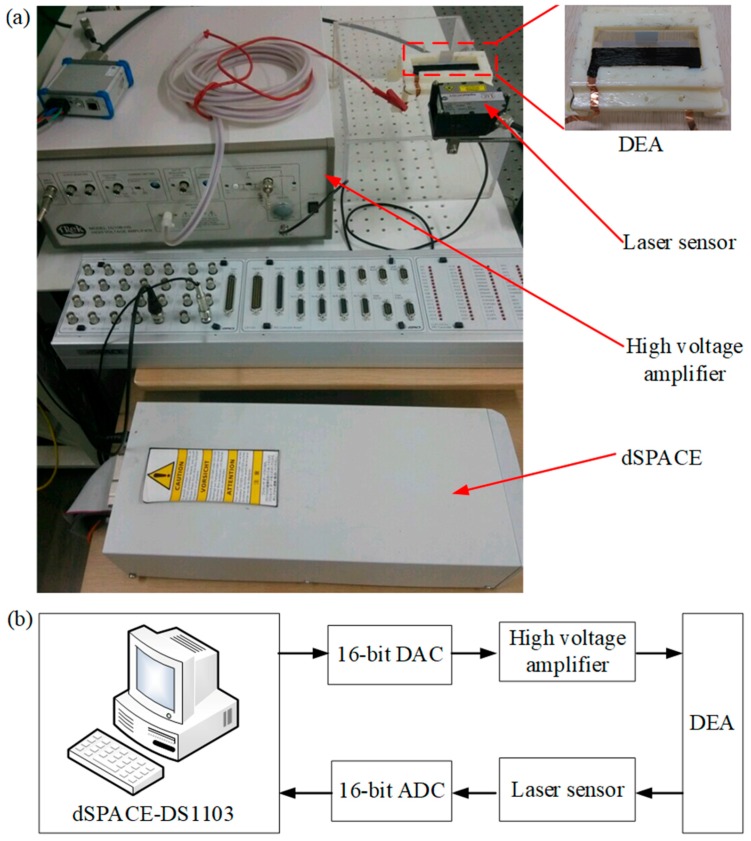
The whole experimental setup: (**a**) experimental platform; (**b**) block diagram.

In order to capture and characterize the viscoelastic hysteresis effect of the planar DEA, we built an experimental setup as shown in Figure 3a. A high-voltage amplifier (TREK 10/10-HS, TREK INC., New York, NY, USA) with a fixed gain of 60 dB was used to apply an exciting voltage for the planar DEA, and a laser sensor (Micro-Epsilon ILD2300-100, range of 0–100 mm with an analogue output of 0–10 V, Micro-Epsilon, Ortenburg, Germany) was utilized to measure the output displacement of the planar DEA. A dSPACE-DS1103 (dsPACE, Paderborn, Germany) board equipped with 16-bit analogue-to-digital converters (ADCs) was adopted to generate a control signal for the high-voltage amplifier and to capture the output signal of the laser sensor. In this work, the sampling time was set to be 1 ms. Figure 3b gives a block diagram of the experimental setup. It should be noted that when the amplitude of the exciting voltage was higher than 3 kV, we could observe several failure phenomena, such as instability and wrinkling [2]. On the other hand, the output displacement of the planar DEA was too small to be observed when the exciting voltage was lower than 0.5 kV. Therefore, we kept the exciting voltage in the range of 0.5–2.5 kV in the study.

### 2.3. Experimental Phenomena

It is known that the viscoelastic hysteresis effect of DEAs is asymmetric and rate-dependent. In order to investigate the behavior of the viscoelastic nonlinearity, we firstly tested the dynamical response of the planar DEA. In this sense, the exciting voltages were selected to be same amplitude with different frequencies, which can be formed as

(1) V(t)=sin(2πft−0.5π)+1.5(kV)

where f represents the frequency of the exciting voltage. It should be noted that a phase delay of 0.5π in Equation (1) was used to reduce the response of the DEA because of the initial input voltage at *t* = 0. Of course, other phase delays could also be selected. Without loss of generality, we selected ϕ=0.5π. In this work, the relationships between the input voltage and displacement were measured under seven randomly selected different frequencies (0.02, 0.05, 0.1, 0.2, 0.4, 0.8, and 1 Hz).

Remark: It is well known that the bandwidth of Very High Bond (VHB) based DEAs is usually limited to 3 Hz. For the specific planar DEA used in this work, the experimental results demonstrate that when the frequency of the exciting voltage increased from 0.02 to 1 Hz, the amplitude of the output displacement monotonously decreased by 27.26%, which means that the working frequency of the planar DEA was limited to about 1 Hz. Therefore, although the maximum effective frequency of the MRPIM was limited to 1 Hz, it could still satisfy the practical application of the planar DEA.

**Figure 4:**
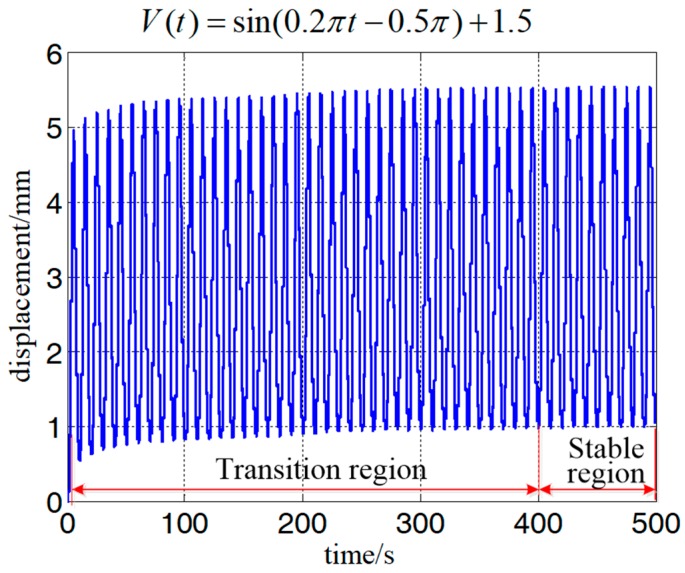
The displacement response of the planar dielectric elastomer actuator (DEA) when the frequency of input voltage equaled 1 Hz.

**Figure 5:**
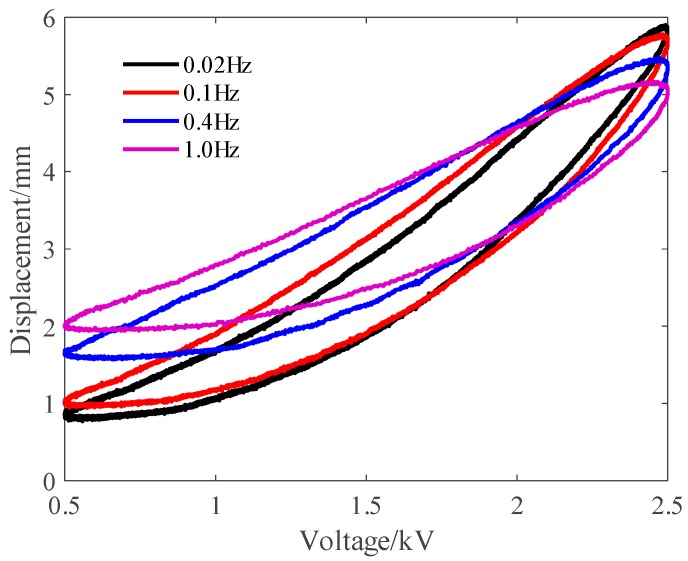
The hysteresis loops under the sinusoidal inputs with different frequencies.

Figure 4 shows the experimental results when the frequency of the exciting voltage equaled 0.1 Hz. As mentioned in Figure 1, the displacement could be separated into a transition region and stable region. During the transition region, a creep effect (slow drift of the displacement [14]) was observed and lasted about 400 s before reaching the stable region. It should be noted that although such creep is caused by viscoelasticity of the DEA, it has been explained by many well-defined models [12,13,14]. Differently from the transition region, the viscoelastic hysteresis effect dominates the dynamical response in the stable region. However, how to model the viscoelastic hysteresis effect of DEAs is still challenging. Therefore, we were motivated to establish a MRPIM to describe the viscoelastic hysteresis effect of DEAs. To avoid the influence of the creep effect, we only took the experimental data of the stable region into consideration for developing the MRPIM. To further analyze the features of the viscoelastic hysteresis effect of the planar DEA, we compared the viscoelastic hysteresis loops with different frequencies (0.02, 0.1, 0.4, and 1 Hz). On the basis of the experimental results shown in Figure 5, we could observe the following:

(i) The viscoelastic hysteresis loops are asymmetric.
(ii) The widths of the viscoelastic hysteresis loops are rate-dependent. When the frequency of the input voltage increased from 0.02 to 1 Hz, the width of the viscoelastic hysteresis loop increased by 21.80%.
(iii) The peak-to-peak displacements of the viscoelastic hysteresis loops are rate-dependent. When the frequency of the exciting voltage increased from 0.02 to 1 Hz, the peak-to-peak displacement of the viscoelastic hysteresis loop decreased by 27.26%.

The above observations demonstrate that the viscoelastic hysteresis loops of the planar DEA are both asymmetric and rate-dependent. As discussed above, physical-based models may be too complicated to describe such viscoelastic hysteresis effects. Therefore, a phenomenological modeling approach was proposed to solve this challenge. At the first step, a MRPIM was developed to characterize the asymmetric and rate-dependent viscoelastic hysteresis effect. Finally, the MRPIM was identified and verified.

## 3. Viscoelastic Hysteresis Model

Classical P–I models have been widely used to describe hysteresis effects mathematically [16,17,22] because of their analytical inversion. The drawback of classical P–I models is that they are generally effective only for symmetric and rate-independent hysteresis effect description. In order to describe asymmetric and rate-dependent viscoelastic hysteresis effects of the DEA, we developed a MRPIM in this work.

### 3.1. The Classical P–I Model

The classical P–I model can be expressed as follows [22]:

(2) $$y(t)=pV(t)+\int_{0}^{R}a(r)F_{r}\left[V\right](t)dr,$$

where the input signal V(t) is any continuous function on the interval $\left[0,t_{E}\right]$, y(t) represents output displacement, p is a positive constant, a(r) is a density function, and $F_{r}\left[V\right]\left(t\right)$ with a threshold r≥0 is a one-side play operator that can be written as follows:

(3) $$\begin{array}{l} F_{r}\left[V\right]\left(0\right)=f_{r}\left(V\left(0\right),0\right)\\ F_{r}\left[V\right]\left(t\right)=f_{r}\left(V\left(t\right),W\left(t_{i}\right)\right) \end{array}$$

for $t_{i}\leq t\leq t_{i+1}$, 1≤i≤N, with

(4) $$f_{r}\left(V,W\right)=\max\left(V-r,\min\left(V,W\right)\right),$$

where $0=t_{1}<t_{2}<\cdots <t_{N}=t_{E}$ is a partition of $\left[0,t_{E}\right]$, such that the function V(t) is a monotone on each of the subintervals $\left[t_{i},t_{i+1}\right]$. Because the polynomial and play operators are rate-independent and symmetric, the classical P–I model cannot describe the asymmetric and rate-dependent viscoelastic hysteresis effect shown in Figure 5. Therefore, it is necessary to develop a new rate-dependent P–I model to describe the viscoelastic hysteresis effect of DEAs.

### 3.2. The MRPIM

The MRPIM can be expressed as

(5) $$y(t)=g\left[V\left(t\right)\right]+q\ddot{V}(t)+\int_{0}^{R}a_{r}F_{or}^{h}\left[V\right]\left(t\right)dr,$$

where y(t) represents output displacement; V(t) and $\ddot{V}\left(t\right)$ are the input signal and its acceleration, respectively; q is a constant ratio; and $g\left[V\left(t\right)\right]=p_{1}V\left(t\right)+p_{2}V{\left(t\right)}^{2}+p_{3}V{\left(t\right)}^{3}+p_{4}V{\left(t\right)}^{4}$ is a polynomial input function with four constants $p_{1},p_{2},p_{3},p_{4}$. The fourth-order polynomial is used to describe the asymmetric behavior. In this work, the order number 4 was selected on the basis of a trial-and-error method, as is generally used in the literature [22,32]. It should be noted that further increasing the order of the polynomial (higher than 4) contributes a little improvement in accuracy but makes the computation costlier. In the following development, we selected the order of the polynomial as 4 for a case study; *a_r_* represents a density function, and $F_{or}^{h}\left[V\right]\left(t\right)$ is the rate-dependent play operator with a constant threshold r that is defined as follows:

(6) $$W({0)=F}_{or}^{h}\left[V\right]\left(0\right)=f_{or}^{h}\left(V\left(0\right),y\left(0\right)\right)\;W(t{)=F}_{or}^{h}\left[V\right]\left(t\right)=f_{or}^{h}\left(V\left(t\right),F_{or}^{h}\left(t-T\right)\right)\;f_{or}^{h}\left(V\left(t\right),W\left(t\right)\right)=\max\left\{h_{l}\left(V\left(t\right),\dot{V}\left(t\right)\right)-r,\min\left(h_{r}\left(V\left(t\right),\dot{V}\left(t\right)\right),W\left(t\right)\right)\right\}$$

where $h_{l}\left(V\left(t\right),\dot{V}\left(t\right)\right)$ and $h_{r}\left(V\left(t\right),\dot{V}\left(t\right)\right)$ are the dynamic envelope functions that depend on the input voltage V(t) and its derivative $\dot{V}\left(t\right)$. On the basis of experimental observation, the dynamic envelope functions can be expressed as follows:

(7) $$h_{l}\left(V\left(t\right),\dot{V}\left(t\right)\right)=V\left(t\right)-\gamma \left|\dot{V}\left(t\right)\right|\;h_{r}\left(V\left(t\right),\dot{V}\left(t\right)\right)=V\left(t\right)+\lambda \left|\dot{V}\left(t\right)\right|$$

where γ and λ are two constants. For the convenience of calculation, $\dot{V}\left(t\right)$ is estimated by [V(t)−V(t−T)]/T, and T is the sampling time.

As usual, the upper limit of the integration is infinite; it is not convenient to identify the parameters of the model, and thus we used a discrete form of the model to replace the integration. The discrete form of the MRPIM can be written as

(8) $$y(t)=p_{1}V(t)+p_{2}V{\left(t\right)}^{2}+p_{3}V{\left(t\right)}^{3}+p_{4}V{\left(t\right)}^{4}+q\ddot{V}(t)+\sum_{i=1}^{N}a_{i}F_{or_{i}}^{h}\left[V\right]\left(t\right),$$

where N is the number of play operators, $a_{i}$ is the weighting factor of the *i*th play operator with a constant threshold value $r_{i}$, and $r_{i}$ is equal to $\left(i-1\right)/N\times {\left\|V(t)\right\|}_{\infty }$, i=1,2,⋯,N.

### 3.3. Identification of the MRPIM

To experimentally verify the effectiveness of our development, the first step was to identify the parameters of the MRPIM, which were obtained by a modified particle swarm optimization (MPSO) algorithm [33]. In this work, the fitness function of the MPSO algorithm was selected as

(9) $$f\left(x\right)=\sum_{m=1}^{4}\left[\frac{k_{m}}{n_{m}}\sum_{i=1}^{n_{m}}{\left(y_{mi}-y_{mi}^{a}\right)}^{2}\right],$$

where m represents the number of the frequency; $n_{m}$ and $k_{m}$ are the values of the input signal and the weight ratio of *i*th frequency, respectively; $y_{mi}$ and $y_{mi}^{a}$ are the predicted results and experimental data at the *i*th sampling time, respectively; and $x=\left\{\gamma ,\lambda ,p_{1},p_{2},p_{3},p_{4},q_{1},a_{1},a_{2},\cdots ,a_{13}\right\}$ is a vector that contains all identified parameters.

Table 1 lists the parameters’ values of the fitness function. MATLAB software was used to perform the identification process, and the identification results are shown in Table 2.

**Figure 6:**
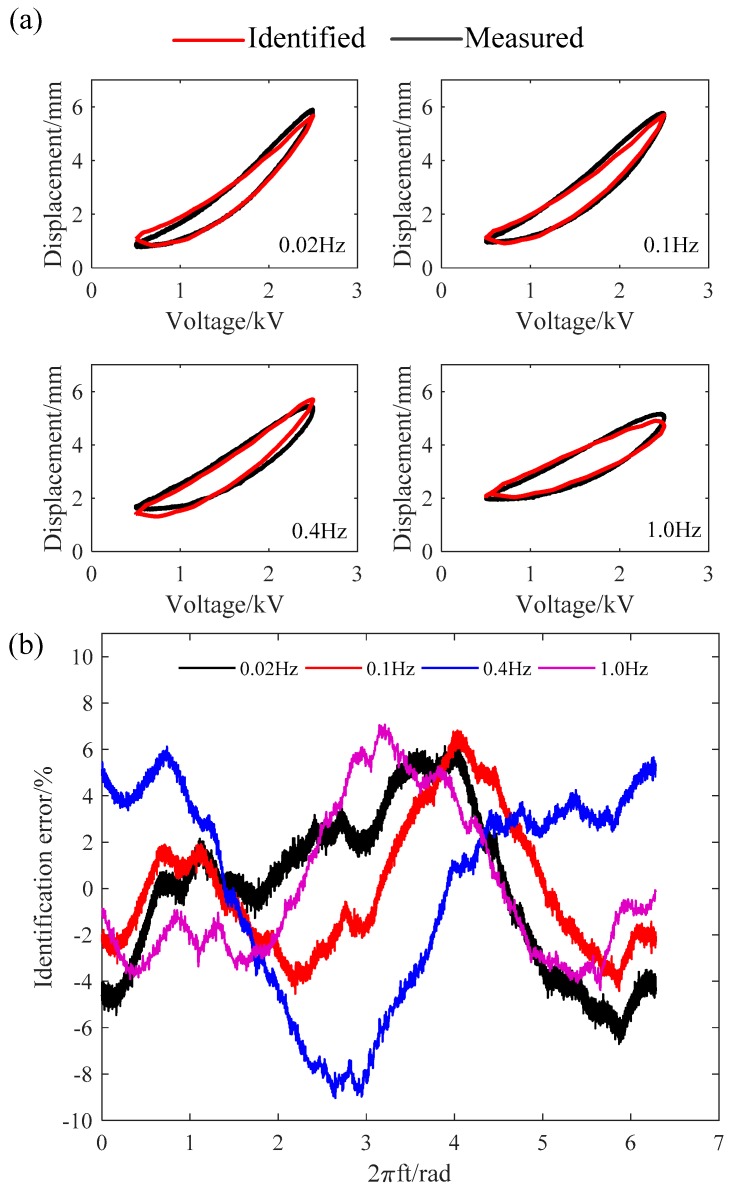
Identification results of the modified rate-depended Prandtl–Ishlinskii model (MRPIM): (**a**) comparisons between identified MRPIM and experimental data; (**b**) the identification errors.

Figure 6a shows the comparisons between the experimental data (black lines) and the identified results (red lines). The identification errors are plotted in Figure 6b. We can see from Figure 6 that the identified MRPIM could accurately describe the hysteresis loops under different frequencies.

To further quantify the performance of the MRPIM, the maximum error $e_{m}$ and the root-mean-square error $e_{rms}$ are defined, respectively, as follows:

(10) $$e_{m}=\max\frac{\left|y(i)-y^{a}(i)\right|}{\max\left(y^{a}\right)-\min\left(y^{a}\right)}\;e_{rms}=\frac{\sqrt{\frac{1}{N}\sum_{i=1}^{N}{\left(y(i)-y^{a}(i)\right)}^{2}}}{\max\left(y^{a}\right)-\min\left(y^{a}\right)}$$

where y(i) and $y^{a}\left(i\right)$ represent predicted results and experimental data, respectively. According to the definitions, the maximum identification error and root-mean-square identification error are listed in Table 3. We found that the maximum identification error and root-mean-square identification error were 9.03% and 4.50%, respectively.

**Figure 7:**
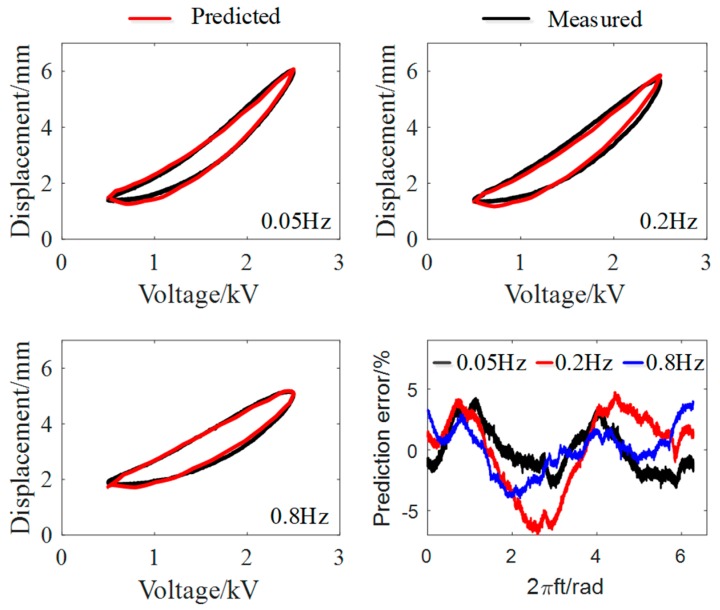
The verification of the modified rate-depended Prandtl–Ishlinskii model (MRPIM).

To further verify the effectiveness of the MRPIM, we used it to predict the viscoelastic hysteresis effect of the planar DEA with three different frequencies (0.05, 0.2, and 0.8 Hz) and compared the predicted results with experimental data. Figure 7 and Table 4 demonstrate that the MRPIM agreed well with the experimental data. The maximum prediction error and the root-mean-square prediction error were 6.95% and 3.25%, respectively. Therefore, the MRPIM can precisely describe the viscoelastic hysteresis effect of the planar DEA when the frequency of the exciting voltage is in the range of 0.02–1 Hz.

## 4. Conclusions

In this work, we propose a phenomenological modeling approach to describe the viscoelastic hysteresis effect of a planar DEA with a MRPIM. Firstly, we investigated the viscoelastic responses of the planar DEA on the basis of different experiments. The experimental results show that (i) the viscoelastic hysteresis loops are asymmetric; (ii) when the frequency of the exciting voltage increased from 0.02 to 1 Hz, the width of the viscoelastic hysteresis loop increased by 21.80%, while the peak-to-peak displacement decreased by 27.26%. Thus, the MRPIM was developed, and the model parameters were identified by a MPSO algorithm. Finally, comparisons between experimental results and MRPIM simulation were performed to verify the effectiveness of the development.

## Figures and Tables

**Table 1:** Parameters of the fitness function.

m	Frequency (Hz)	$k_{m}$	$n_{m}$
1	0.02	4	50,000
2	0.1	1	10,000
3	0.4	4	2500
4	1	1	1000

**Table 2:** Identification result of the modified particle swarm optimization (MPSO) algorithm.

i	$a_{i}$	$p_{i}$	q	γ	λ
1	−4.3668	−2.0536	0.0254	0.0323	−0.0322
2	0.7119	5.5811			
3	−0.7480	−1.5452			
4	0.5184	0.1641			
5	−0.5119				
6	0.3491				
7	−0.0132				
8	0.5967				
9	0.3318				
10	0.7624				
11	2.7350				
12	9.9937				
13	−12.2643				

**Table 3:** Identification errors at different frequencies.

Frequency (Hz)	$e_{m}$ (%)	$e_{rms}$ (%)
0.02	6.70	3.40
0.1	6.80	2.82
0.4	9.03	4.50
1.0	7.07	3.28

**Table 4:** Prediction errors at different frequencies.

Frequency (Hz)	$e_{m}$ (%)	$e_{rms}$ (%)
0.05	4.27	1.82
0.2	6.95	3.25
0.8	4.01	1.85
